# Clinical Features and Outcome of Sporadic Colorectal Carcinoma in Young Patients: A Cross-Sectional Analysis from a Developing Country

**DOI:** 10.1155/2014/461570

**Published:** 2014-04-01

**Authors:** Muhammad Nauman Zahir, Eisha Mahpara Azhar, Sobia Rafiq, Kulsoom Ghias, Munira Shabbir-Moosajee

**Affiliations:** ^1^Department of Oncology, Aga Khan University Hospital, Stadium Road, P.O. Box 3500, Karachi 74800, Pakistan; ^2^Department of Biological and Biomedical Sciences, Aga Khan University Hospital, Stadium Road, P.O. Box 3500, Karachi 74800, Pakistan

## Abstract

*Background*. Early onset colorectal carcinoma (CRC) is rare and has been hypothesized to be a biologically and clinically distinct entity personifying aggressive disease and worse survival. *Methods*. Data for 131 patients was collected by retrospective chart review. Cox proportional hazard model was used to compute prevalence ratios and 95% confidence intervals. *Results*. Early onset sporadic CRC accounted for 32% of all CRC treated in the specified time period. The mean age was 33.3 ± 7.9 years and the male to female ratio was 2 : 1. Colon and rectal cancers accounted for 55% and 45% of patients, respectively. 96% of rectal carcinoma patients received appropriate therapy as opposed to 65% of colon cancers. On multivariable analysis, appropriate reception of therapy (PR 4.99; 95% CI, 1.21–20.6) and signet ring morphology (PR 2.40; 95% CI, 1.33–4.32) were significantly associated with rectal cancers as opposed to colon cancer. Kaplan-Meier analysis revealed a trend towards inferior survival for rectal carcinoma 2 years after diagnosis. *Conclusion*.A high prevalence of early onset CRC was noted in the study. A trend towards inferior survival was seen in patients with rectal cancer. This finding raises the possibility of rectal carcinoma being an aggressive subset of young CRC.

## 1. Introduction

Colorectal carcinoma (CRC) is one of the most common cancers diagnosed worldwide. It has been documented that CRC is the second most common malignancy in females and the third most common amongst males [1]. Epidemiological data suggests a higher incidence of sporadic CRC in developed countries, with Australia and New Zealand having an age adjusted incidence of 45.7 per 100,000 as compared to Southeast Asia which has a reported incidence of 15.9 per 100,000 [1]. Interestingly, a rapid increase in CRC incidence rate in economically transitioning countries has been recently reported in the literature [2] and has been attributed to a change in the dietary habits and physical activity patterns superimposed on genetic predisposition.

Age is considered a major risk factor for colon cancer. Diagnosis of CRC is a rare occurrence in patients less than 40 years. Thereafter, the incidence increases sharply with every decade of life. In developed countries, the risk of colon cancer is 1 in 10 males after the 8th decade of life [3]. The life time risk of sporadic CRC is 5% and it accounts for 90% of the cases diagnosed in patients above 50 years [3].

Early onset CRC accounts for around 7% of the total CRC population in the West [4] but the problem has been reported to be of a much greater magnitude in several Asian and African countries [5–12]. Similarly, half of the incident cases of CRC in the Pakistani population have been estimated to be in young patients [13]. From this, coupled with the fact that greater than 80% of the Pakistani population is younger than 40 years [14], the “at-risk” population for early onset CRC in countries like Pakistan is much higher than the rest of the world.

Studies from the past have shown that in low incidence countries like Pakistan, CRC tends to be located in the distal colon and rectum while it is more likely to be located proximally in countries where incidence is higher [15]. Furthermore, dominance of left sided tumors in the young population has also been demonstrated in other studies [4]. This finding is consistent with our clinical experience where we see a large proportion of rectal cancers in our young CRC population. The exact proportion of this subgroup of patients presenting with distally located disease has however never been reported from our region.

It has been hypothesized that early onset colorectal cancer is a biologically and clinically distinct disease where younger individuals have more aggressive disease and worse survival [16, 17]. A recent population based study from Pakistan also showed that younger patients presented with disease which was more likely to be poorly differentiated and at a more advanced stage at diagnosis [13]. Although there have been a few studies regarding local experience in treatment and management of colon cancer in the past, data pertaining specifically to early onset colorectal cancer is lacking. Given the increased incidence of early onset CRC in Pakistan and the greater “at-risk” population, this study was undertaken to report the clinical and pathological characteristics along with the outcome of this unique subgroup of patients through data from a tertiary care oncology center in Karachi, Pakistan.

## 2. Methods

### 2.1. Patients

We retrospectively collected data of patients less than or equal to 45 years of age with newly diagnosed CRC, presenting to the Oncology Department, Aga Khan University Hospital (AKUH), Karachi, between January 1, 2004, and December 31, 2011. Patients were identified using the discharge coding system. Patients with suspected familial syndromes (based upon clinical history and colonoscopic findings) incompletely documented histopathological reports and with biopsy reports from other centers were excluded from analysis.

The data was collected through chart review using a predesigned and coded questionnaire which had been approved by the institutional Ethical Review Committee. Details of the demographics, symptomatology, risk factors, family history, histopathological features, and outcome were collected for all patients. Histopathology and surgical records were reviewed for tumor location, tumor grade, dissected lymph nodes, involved lymph nodes, and presence of lymphovascular invasion and perineural invasion. The American Joint Committee on Cancer Staging System (AJCC 7th edition) was used to stage the disease.

Follow-up records, data pertaining to therapies, and dates of last contact were also recorded. Patients who had not followed up for greater than 6 months were contacted on the telephone numbers obtained from the hospital database. Any additional information regarding outcome accrued through this telephonic contact was incorporated into the recorded data. A patient was labeled “lost to follow-up” if all attempts to establish contact with him/her failed.

### 2.2. Statistical Analysis

Data pertaining to 131 patients, who were found eligible for the study, were analyzed. Statistical Package for Social Sciences (SPSS) version 19 was used to perform data analysis which was reported as mean ± standard deviation for continuous variables and proportions and percentages for categorical data. Continuous variables not following the normal distribution were reported as medians along with interquartile ranges. Analysis was performed on the entire cohort followed by a subgroup analysis according to anatomical location of the tumor (colon versus rectal). Kaplan-Meier curves were generated for median overall survival (OS) for both colon and rectal carcinomas which were compared using the log-rank test.

Univariate and multivariable analysis was performed using the Cox proportional hazard model which was used to compute prevalence ratios and 95% confidence intervals. *P* value less than 0.05 was considered significant.

## 3. Results

### 3.1. Patients

A total of 581 patients presented with newly diagnosed CRC to our centre during the study period. Of these 186 (32%) were younger than 45 years and 131 were found to be eligible for analysis. Most of the patients were diagnosed via a colonoscopy (83%), while 97 patients (74%) had advanced disease, defined as stage III/IV, at initial presentation.

The mean age of the study population was 33.3 ± 7.9 years. Male to female ratio was 2 : 1. No significant genetic predisposing factors (such as familial adenomatous polyposis, Lynch syndrome) were suspected in included patients. Only 8 patients (6.1%) had a positive family history of colon cancer but were assumed to have sporadic CRC as they did not fulfill the Amsterdam criterion for hereditary nonpolyposis colorectal carcinoma (HNPCC) or the clinical criteria for familial adenomatous polyposis (FAP). The most common clinical presentations were abdominal pain (87%, *n* = 114), weight loss (66%, *n* = 86), and bleeding per rectum (52%, *n* = 68). Only 29 patients (22%) presented with intestinal obstruction.

55% of the cohort (*n* = 72) had colon cancer while the rest had presented with a diagnosis of rectal carcinoma (*n* = 59). 38 patients (53%) with colon cancer had right sided disease. The colon and rectal cancer groups were similar in age of presentation and sex. However, bleeding per rectum was much more common in rectal carcinomas (85% versus 25%), whereas patients with colon cancer were more likely to present with an abdominal lump (17% versus 7%) and intestinal obstruction (29% versus 14%) and were more likely to have preexisting polyps (13% versus 2%) (Table 1).

### 3.2. Histopathological Data

On histopathology, preexisting polyps were identified in only 10 patients (7.6%). 73 patients (56%) had moderately differentiated carcinoma and further 50 patients (38%) had poorly differentiated carcinoma. Signet ring morphology was identified in 28 patients (21%), perineural invasion in 21 (22%), and lymphovascular invasion in 25 (26%) of surgical specimens. 76% of patients undergoing curative surgery had undergone appropriate lymph node dissection (>12 nodes).

When right sided colon cancers were compared to left sided colon cancers, no statistically significant differences were found in histopathological characteristics between these two groups.

On the other hand, there was a greater preponderance of poor prognostic features such as poorly differentiated carcinoma (49% versus 29%), signet ring morphology (37% versus 8%), and advanced stage (80% versus 69%) in the rectal carcinoma group as compared to patients with disease of the colon.

### 3.3. Treatment

92 patients (70%) were treated with curative intent, while the rest had presented with metastatic disease at diagnosis and received palliative treatment. Half of the patients treated with curative intent had rectal cancer.

**Figure 1:**
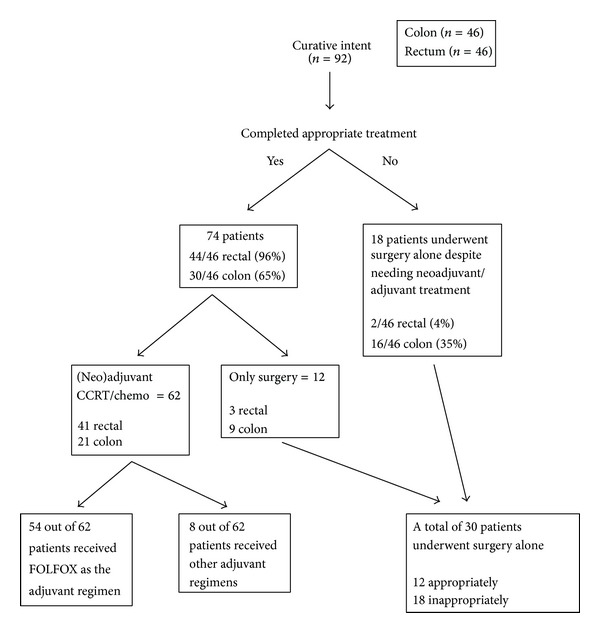
Flowchart of patients treated with curative intent.

The rectal carcinoma group had a higher percentage of patients who completed appropriate therapy (96% versus 65%) (Figure 1). FOLFOX (oxaliplatin, calcium leucovorin, and 5-fluorouracil) was used as first line adjuvant treatment in 87% of patients (*n* = 54) who received adjuvant therapy (Figure 1).

### 3.4. Outcome Analysis

At the time of analysis, 61 patients (47%) had been lost to follow-up either during or after completion of treatment of which 36 (59%) were in remission and 22 (41%) had progressive disease. Of the remaining 70 patients (53%) who had maintained regular follow-up, 38 patients (54%) had died, 30 (43%) were in remission, and further 2 (3%) were still undergoing treatment.

**Figure 2:**
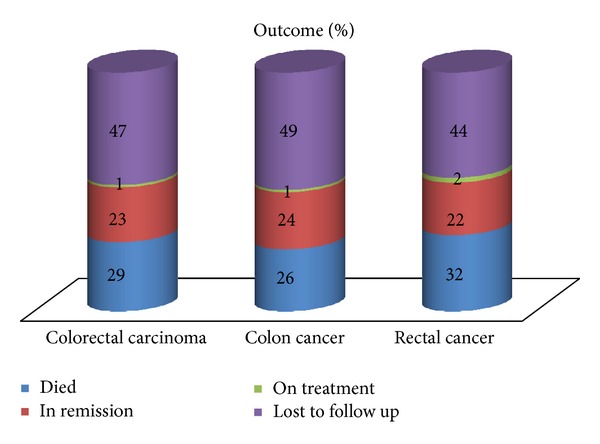
Outcome of the study population.

A higher proportion of patients with colon carcinoma were lost to follow-up when compared with the rectal carcinoma cohort (49% versus 41%). Of the patients who maintained regular follow-up, a higher proportion of patients were found to be in remission in the colon cancer group (46% versus 39%). Conversely, a higher proportion of patients with rectal carcinoma had died at the time of analysis (58% versus 51%) (Figure 2).

The median time to progression for the 60 patients (45.8%) who had either died or were progressing at last follow-up was 8.25 months (IQR = 3.6–16.3 months). This figure was 9 months for the patients with colon cancer (*n* = 27) and 7.5 months for the patients with rectal carcinoma (*n* = 33). The median progression-free survival (PFS) in the 2 groups was not statistically different (*P* = 0.70).

**Figure 3:**
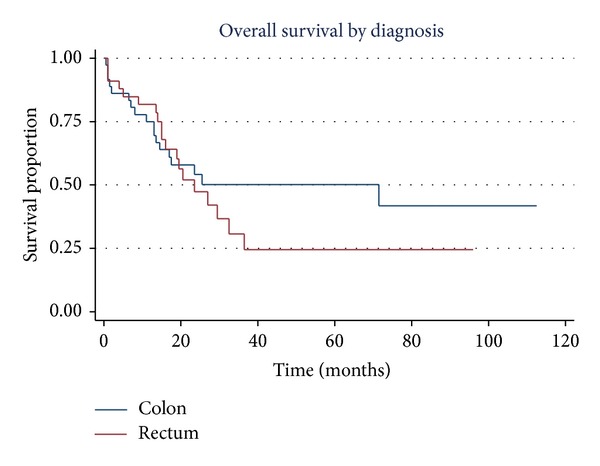
Median overall survival for colon and rectal carcinomas.

Median OS for the entire cohort was 19 months (IQR = 12.6–33.6 months). The median OS was similar between the right sided and left sided colonic cancer groups and was also not statistically different between the cohorts of colon cancer and rectal carcinoma (*P* = 0.58). Kaplan-Meier analysis, however, revealed a trend towards an inferior survival for rectal carcinoma 2 years after initial diagnosis (Figure 3).

On univariate analysis signet ring morphology, presence of polyps, appropriate reception of therapy, poorly differentiated tumor, and late stage at presentation were found significantly different between the two groups (Table 2). However, on multivariable analysis, only appropriate reception of therapy (PR 4.99; 95% CI, 1.21–20.6) and signet ring morphology (PR 2.40; 95% CI, 1.33–4.32) remained significantly associated with rectal cancers as opposed to colon cancer (Table 2).

## 4. Discussion

The definition of “early onset” CRC varies in the literature. Most have defined “early onset” as <40 years though upper limits ranging from 30 to 50 years have been used [18]. Epidemiological surveys indicate that the incidence of colorectal cancers is much lower in the developing countries. Conversely, there have been reports that suggest a higher incidence of early onset disease in the developing world [19]. The exact cause of this difference is not known; however, it can be postulated that the higher proportion of younger patients in the underdeveloped countries can be a reason for this skewed finding [16]. However, more interesting is the hypothesis that sporadic early onset colorectal cancer is a biologically and clinically distinct entity, accounting for its aggressive presentation and poorer survival [16, 17, 20].

In a meta-analysis, the proportion of young CRC patients (less than 40 years) among all CRC patients ranged from 0.4% to 36.5% with an average of 7% [4]. The majority of these studies were conducted in North America and Europe. The results of this meta-analysis and several other studies indicate that sporadic early onset colon cancer is rare in the developed countries. On the other hand, early onset CRC is far more common in third world countries [8, 13]. We report a prevalence of sporadic early onset CRC of 32% at our institution during the study period which is substantially higher than what western data suggests [4]. This is also higher than estimates from other Asian studies [7, 16, 21], although it was comparable to that from India [8, 22].

There are several potential explanations for this interesting observation. First of all this study is an institution based study where selection bias cannot be avoided. Moreover, a tertiary care center such as ours would ensure greater concern and provide better facilities for cancer detection than those available generally in the country. This may result in inflation of the prevalence results derived. Secondly, the presence of a very large proportion of the population which is “young” [6] increases the number of “at-risk” subjects and may be responsible for a larger number of cases.

A large collection of available data suggests that several dietary and lifestyle factors may significantly contribute to the risk of development of CRC [23]. Of these, physical inactivity, excess body weight, and central deposition of adiposity are established risk factors [23] and probably play an prominent role in the pathogenesis of the disease in our population where the prevalence of these factors is unacceptably high [5]. Westernization of the diet with resultant overconsumption of energy and developing insulin resistance may contribute to the prevalence in the more affluent sector of the country's population, while the lack of specific macronutrients and certain other dietary ingredients in meals may play a part in the under privileged strata [23]. Smoking early in life, which is another established risk factor for the development of colon cancer, may also play a part in the high incidence observed at our institution with almost a quarter of young adults in the country (aged 18–25) being current smokers [24]. The fact that data on these lifestyle factors could not be collected in our study secondary to the retrospective nature of data collection highlights one of our limitations.

Interestingly, SEER (surveillance, epidemiology, and end results) database analysis from 1998–2002 indicates that there was a higher proportion of patients with early onset CRC in Asians of Indian and Pakistani origin, despite residing in USA for many years [25]. This finding points to a possible genetic or epigenetic etiology for development of early onset CRC in this specific population bearing in mind the fact that chromosomal instability (CNI) and microsatellite instability (MSI) do occur in sporadic cases of CRC [26, 27]. The established increased propensity of developing a colorectal malignancy in this particular subgroup of patients has led to calls for revision of screening guidelines in the Asian population to include the high risk younger age groups [13], but current guidelines remain almost identical to those adapted by the developed countries [28].

An almost equal prevalence of colon and rectal cancer that we found concurred with the ratio seen in countries which have historically been labeled as “low risk” [13, 29]. A worse stage at presentation, poorer grade of tumor, and an increased prevalence of signet ring morphology in our study population were similar to those reported globally reflecting the aggressive nature of the disease in young CRC patients [4, 30, 31].

The fact that only 80% of patients who were planned for curative therapy received appropriate therapy (surgical/neoadjuvant/adjuvant) is a matter of concern as it leaves this young population at a higher risk for recurrence in the future. The problem can be attributed to the fact that Pakistan is a resource-limited country with most of the health expenditure derived from out-of-pocket payment and the financial burden incurred as a result translates into poor adherence to standard treatment guidelines in most defaulting cases.

The median OS for our cohort of young CRC patients was 19 months. Studies from neighboring countries with similar socioeconomic status such as Nepal and India have shown a dismal survival for young CRC patients [11, 32]. As we would expect, the survival data is varied globally and some studies have suggested that younger age either had no effect or a positive effect on survival [4, 31, 33–35]. However, this is debatable and needs to be taken in context with the geographical location, tumor biology, and appropriateness of treatment.

It is important to bear in mind that our poor survival results may have been exaggerated to a certain extent secondary to the substandard follow-up of some of our patients and the high lost to follow-up rate witnessed in our study when compared to standards of the West and the well-developed Asian countries. This is a major limitation of all the data that comes out of Pakistan as patients in our country are not supported by the state or insurance. In a country where the gross national income per capita is only $1,050 [36], patients are commonly faced with difficult choices and usually opt not to pursue regular follow-up and investigational visits especially if deemed “cured.” It can be extremely challenging to stay in touch with these patients as a significant percentage do not have telephonic access as well. This leaves us with incomplete data pertaining to our patients and potential exaggeration of the poor survival we report due to missing information on “survivors.”

During the course of the study, an interest developed to compare the subgroups of the disease (colon versus rectal). This idea was instigated by a previous study which concluded that distinct mechanisms of oncogenesis may be responsible for tumors of the rectum [37]. This subgroup analysis revealed that signet ring morphology of the tumor cells and poorly differentiated carcinomas, which have historically been regarded as poor prognostic factors [4, 30, 31], were significantly more prevalent in the rectal group. Another interesting fact that was highlighted was the statistically significant proportion of rectal cancer patients completing appropriate therapy as opposed to patients with colon carcinoma. This difference probably arises from the chronology of treatment for the two subgroups whereby rectal carcinoma patients receive neoadjuvant therapy, whereas patients with colon cancer undergo surgery first giving them a false sense of security of having achieved “cure” and dampening their motivation of pursuing adjuvant treatment.

Although the median OS was not statistically different between the two subgroups, analysis revealed a trend towards inferior prognosis for rectal carcinoma 2 years after initial diagnosis. This, despite the fact that a higher number of patients in this same subgroup completed appropriate therapy, raises the possibility of rectal carcinoma being an aggressive subset of the young CRC population. Further weight is added to this hypothesis by studies conducted in the past which have shown significant difference in survival between colon and rectal cancers [38, 39]. A Turkish study in particular documented a 5-year survival rate of 81% for colon cancer which was more than double that for rectal carcinoma (39%) in patients less than 40 years of age [38]. This opens doors to evaluate differences in genetics, biology, severity, and prognosis of rectal cancers from colon cancers in general.

The strengths of our study include the fact that this is the first report of young CRC patients from the country. It is also unique in that it compares the different aspects of the 2 subgroups of CRC including the completion of appropriate treatment and provides us with an estimate of the survival of these patients, data regarding which is extremely scarce from our region.

## 5. Conclusion

We conclude that a high incidence of early onset CRC is noted in our study population of which almost half had rectal disease. This group had a higher prevalence of poor prognostic factors and showed a trend towards inferior prognosis 2 years after diagnosis despite the fact that a larger proportion of these patients had completed appropriate therapy. These findings raise the possibility of rectal carcinoma being an aggressive subset of the young colorectal carcinoma population. Further investigation is required to test this hypothesis.

Our results indicate that young CRC is a distinct entity which should be identified early, possibly by modifying screening guidelines in countries where incidence of early onset colorectal cancer is high. Bench research is also required to elucidate the genetic, pathophysiological, and molecular characteristics of this high risk population whereby early detection could be made through molecular testing and the management modified accordingly.

## Figures and Tables

**Table 1:** Baseline characteristics of the study population.

Variables	All colorectal carcinoma (*n* = 131)	Colon cancer (*n* = 72)	Rectal cancer (*n* = 59)
Age, mean (SD)	33.3 (7.9)	33.74 (8.0)	32.80 (8.0)
16–25 years (%)	24 (18)	12 (17)	12 (20)
26–35 years (%)	44 (34)	24 (33)	20 (34)
36–45 years (%)	63 (48)	36 (50)	27 (46)
Gender, male (%)	87 (66.4)	50 (69.4)	37 (62.7)
Bleeding P/R (%)	68 (51.9)	18 (25)	50 (84.7)
Abdominal lump (%)	16 (12.2)	12 (16.7)	4 (6.8)
Intestinal obstruction (%)	29 (22.1)	21 (29.2)	8 (13.6)
Stage at diagnosis (%)			
Stages 1 and 2	34 (26)	22 (31)	12 (20)
Stages 3 and 4	97 (74)	50 (69)	47 (80)
Grade of tumor (%)			
Grade 1	8 (6.1)	4 (5.6)	4 (6.8)
Grade 2	73 (55.7)	47 (65.3)	26 (44.1)
Grade 3	50 (38.2)	21 (29.2)	29 (49.2)
Presence of signet ring cell morphology (%)	28 (21)	6 (8)	22 (37)
Presence of polyps (%)	10 (7.6)	9 (12.5)	1 (1.7)

**Table 2:** Univariate and multivariable analysis.

Variable	Univariate analysis		Multivariable analysis	
	PR	CI	PR	CI
Received appropriate treatment	5.35	1.30–22.1	4.99	1.21-20.6
Signet ring morphology	2.19	1.29–3.71	2.40	1.33-4.32
Poorly differentiated tumor	1.56	0.94–2.61	—	
Stage III or IV at presentation	1.37	0.73–2.59	—	
Presence of polyps	0.21	0.03–1.50	—	

## Abbreviations

CRC: Colorectal carcinoma
AKUH: Aga Khan University Hospital
AJCC: American Joint Committee on Cancer
SPSS: Statistical Package for Social Sciences
HNPCC: Hereditary nonpolyposis colorectal carcinoma
FAP: Familial adenomatous polyposis
OS: Overall survival
FOLFOX: Oxaliplatin, calcium leucovorin, and 5-fluorouracil
IQR: Interquartile range
PFS: Progression-free survival
PR: Prevalence ratio
CI: Confidence interval
SEER: Surveillance, epidemiology, and end results
CNI: Chromosomal instability
MSI: Microsatellite instability.
